# Electrochemically Enhanced Delivery of Pemetrexed from Electroactive Hydrogels

**DOI:** 10.3390/polym14224953

**Published:** 2022-11-16

**Authors:** Sophie Au-Yong, Melike Firlak, Emily R. Draper, Sofia Municoy, Mark D. Ashton, Geoffrey R. Akien, Nathan R. Halcovitch, Sara J. Baldock, Pierre Martin-Hirsch, Martin F. Desimone, John G. Hardy

**Affiliations:** 1 Department of Chemistry, Faraday Building, Lancaster University, Lancaster LA1 4YB, UK; 2 Department of Chemistry, Gebze Technical University, Gebze 41400, Turkey; 3 School of Chemistry, Joseph Black Building, University of Glasgow, Glasgow G12 8QQ, UK; 4 Instituto de Química y Metabolismo del Fármaco (IQUIMEFA), Facultad de Farmacia y Bioquímica, Consejo Nacional de Investigaciones, Científicas y Técnicas (CONICET), Universidad de Buenos Aires, Junín 956, Piso 3° (1113), Buenos Aires 1113, Argentina; 5 Lancashire Teaching Hospitals NHS Trust, Royal Preston Hospital, Sharoe Green Lane, Preston PR2 9HT, UK; 6 Materials Science Institute, Lancaster University, Lancaster LA1 4YB, UK

**Keywords:** hydrogels, stimuli-responsive, biomedical engineering, drug delivery

## Abstract

Electroactive hydrogels based on derivatives of polyethyleneglycol (PEG), chitosan and polypyrrole were prepared via a combination of photopolymerization and oxidative chemical polymerization, and optionally doped with anions (e.g., lignin, drugs, etc.). The products were analyzed with a variety of techniques, including: FT-IR, UV-Vis, ^1^H NMR (solution state), ^13^C NMR (solid state), XRD, TGA, SEM, swelling ratios and rheology. The conductive gels swell ca. 8 times less than the non-conductive gels due to the presence of the interpenetrating network (IPN) of polypyrrole and lignin. A rheological study showed that the non-conductive gels are soft (G′ 0.35 kPa, G″ 0.02 kPa) with properties analogous to brain tissue, whereas the conductive gels are significantly stronger (G′ 30 kPa, G″ 19 kPa) analogous to breast tissue due to the presence of the IPN of polypyrrole and lignin. The potential of these biomaterials to be used for biomedical applications was validated in vitro by cell culture studies (assessing adhesion and proliferation of fibroblasts) and drug delivery studies (electrochemically loading the FDA-approved chemotherapeutic pemetrexed and measuring passive and stimulated release); indeed, the application of electrical stimulus enhanced the release of PEM from gels by ca. 10–15% relative to the passive release control experiment for each application of electrical stimulation over a short period analogous to the duration of stimulation applied for electrochemotherapy. It is foreseeable that such materials could be integrated in electrochemotherapeutic medical devices, e.g., electrode arrays or plates currently used in the clinic.

## 1. Introduction

Biologically active compounds have been used for several thousand years to prolong and improve life (e.g., pain relief, vaccines, etc.) [1,2]. It is important to note that the biological activity of drugs can be reduced before they reach the desired cells due to degradation via enzymes and low absorption rates, and polymeric matrices can enhance the delivery of such compounds. Drug delivery via needles/tablets/etc. tends to result in burst release profiles and undesired side effects due to the delivery of the bioactive to otherwise healthy cells/organs/tissues [3], and systems capable of precisely controlling the delivery of compounds offer opportunities for innovation in healthcare systems worldwide (e.g., improving patient compliance, minimizing unwanted side effects, etc.) [3,4]. Stimuli-responsive drug delivery systems that can trigger the release of their payloads upon exposure to bio-/physico-chemical cues (e.g., enzymes [5], light [6], pH [7], temperature [8]), are capable of releasing a controlled amount of drug when desired.

Electrically triggered release systems offer a high degree of control of quantity/rate of release, potential for integration in existing medical devices (e.g., electrodes for sensing/stimulation, implants, etc.) and with instrumentation capable of controlling the delivery systems [9]. Such delivery systems can be easily miniaturized, and controlled/powered remotely [9,10], or indeed using sacrificial power sources [11], in various materials morphologies [12]. Polypyrrole (PPy) is one of the most widely investigated conducting polymers applied to drug delivery matrices due to its biocompatibility, low voltage actuation and ease of synthesis [13]. A variety of other conducting polymers (including polyaniline (PANI) and poly(3,4-ethylenedioxythiophene) (PEDOT)) have been investigated for their application in the development of controlled drug delivery systems [14,15]. The drug/biomolecule loading and release process depends on electrostatic interactions between the electroactive polymer and charged molecules which can be tuned by modulating the applied voltage and time of the electric stimulation, with potential for incorporation of low and high molecular weight drugs [10].

Polymer-based hydrogels are popular for biomedical applications, particularly in the development of tissue scaffolds and drug delivery systems because the polymer networks are swollen with water [16,17] facilitating transport of nutrients/waste from tissue scaffolds and bioactive payloads for drug delivery systems [18,19]. The appeal is also driven by the variety of polymers available (particularly PEG [20,21]) methods for the preparation of 3D hydrogels (e.g., chemical/photochemical/in situ crosslinking) and ease with which it is possible to tailor the properties (e.g., pore size distribution, swelling ability, mechanical properties, etc.) of the resulting hydrogels to meet the requirements of specific biomedical applications [22,23].

Polysaccharides are a class of abundant biopolymers [24,25] that are degradable (e.g., enzymatically/hydrolytically) [26], non-immunogenic [27], and a variety of polysaccharide-based biomaterials have been clinically translated [28,29,30]. Chitosan (CS) is one example of a polysaccharide, which is a partially deacetylated derivative of chitin, which displays amines [31] that can be used to facilitate crosslinking, either in situ or to attach other polymerizable species to (e.g., methacrylates) [32]. CS has been widely used in the development of hydrogel-based biomaterials [33,34,35]. Lignins are another class of abundant biopolymers that display antibiotic and anti-fungal activity [36,37], which motivates their inclusion in biomaterials.

Conducting polymers are a class of stimuli-responsive polymers that have potential for use in biomedical applications (e.g., neural electrodes, drug delivery systems, etc.) [38,39] and technical applications (e.g., diodes, sensors, transistors, etc.) [40,41,42], and are therefore interesting for integration in gels. Conducting polymers (e.g., polyaniline (PANI), polypyrrole (PPy) and poly(3,4-ethylenedioxythiophene) (PEDOT)) have been implanted in vivo for long periods of time with minimal inflammatory response (analogous to FDA-approved poly(lactic-co-glycolic acid), PLGA) [43], which can be integrated into existing medical devices [44,45], and it is possible to generate degradable versions of them [46]. Doping conducting polymers with high molecular weight may tune the mechanical properties of the materials [47,48] or indeed cell responses to the surfaces of the biomaterials [49]. Conducting polymer-based drug delivery systems respond to electrical stimuli and function by mechanisms including actuation, charge passage, redox switching, etc. [50] enabling precise spatiotemporal control of the amount of drugs of various molecular weights [51,52,53,54,55].

Conducting polymer-based electrode coatings enable delivery of drugs in the proximity of the site at which the electrodes are implanted, which is potentially impactful for electrochemotherapy which functions by electroporation of the cancerous tissue shortly after injection of anticancer drugs such as bleomycin or cisplatin [56,57,58,59,60]. Electrochemotherapy functions through a variety of mechanisms, including: the cytotoxic effect of the chemotherapeutic which is enhanced by electroporation; vasoconstriction of tumor blood vessel for several hours due to sympathetic nerve stimulation which reduces drug washout from the tumor; vascular disruption due to cytotoxicity towards the endothelial cells of tumor blood vessels; the immune response to immunogenic cell death and enhanced tumor antigen expression [61,62,63]. Electrochemotherapy has been used to enhance the delivery of a variety of drugs [59,60,64,65,66,67,68], and has been observed to be a swift, safe and effective treatment method for cancers that tends to enhance the quality of life of patients [69,70,71,72,73].

Here, we describe the development of electroactive hydrogels composed of PEG, CS, PPy and lignin (serving as both a reinforcing agent and dopant for PPy) that were produced via a combination of photopolymerization and oxidative chemical polymerization; the PPy in the hydrogels was doped with anions (e.g., lignins, drugs). The materials were analyzed by a variety of techniques including: FT-IR, UV-Vis, ^1^H NMR (solution state), ^13^C NMR (solid state), SEM, XRD, TGA, swelling ratios and rheology. In silico and in vitro methods were employed to validate the potential application of the materials for biomedical applications, exemplified by drug delivery studies for the FDA-approved chemotherapeutic pemetrexed (PEM). Such hydrogels could be used as coatings for medical devices, for example, as coatings for electrodes used for electrochemotherapy for cancer treatment [74,75], wherein triggered release of an anticancer drug directly into the cancerous tissue would potentially be more effective and diminish unwanted side effects enhancing the patient’s quality of life.

## 2. Materials and Methods

Unless otherwise noted, all chemicals and consumables were purchased from either Sigma Aldrich (St. Louis, MO, USA) or Thermo Fisher (Waltham, MA, USA), of analytical grade, and used without further purification/modification.

### 2.1. Synthesis

#### 2.1.1. Preparation of Polymerizable CS Derivatives

CS (2 g, medium molecular weight, estimated 75–85% deacetylation) was added to water (200 mL), to which pyrrole-2-carboxylic acid (1 g, 9 mmol) and methacrylic acid (0.76 mL, 0.78 g, 9 mmol) was added, and the mixture was stirred vigorously until the CS dissolved. *N*-(3-dimethylaminopropyl)-*N*′-ethylcarbodiimide hydrochloride (EDC, 3.8 g, 19.8 mmol) and *N*-hydroxysuccinimide (NHS, 2.8 g, 19.8 mmol) were added to the solution by stirring for 24 h at room temperature in the dark. The modified CS was purified by dialysis (MWCO 3500) against deionized (DI) water for 3 days (changing the water at least 4 times per day), followed by lyphophilization for 4 days. The pink/purple solid product was isolated in a quantitative yield (2.1 g). This was combined with other components to prepare hydrogels using the formulations in Table 1.

#### 2.1.2. Preparation of Non-Conductive Hydrogels

A solution of modified CS in DI water (0.45 wt%) was stirred until homogenous. The photoinitiator 2-hydroxy-4′-(2-hydroxyethoxy)-2-methylpropiophenone (Irgacure-2959) was added at 0.1 wt% to the solution of CS derivative; and PEG-diacrylate (PEGDA, Mn 2000) was added at 5 wt%. This mixture was stirred in the dark for ca. 2 h until homogenous. Silicone isolators (molds) produced by Grace Bio-Labs (Sigma Aldrich) (9 mm in diameter × 0.8 mm in depth) were placed onto clean glass slides. 75 μL of gel precursor solution was added to each well and irradiated for 1 h under a Mega LV202E UV exposure unit (with 2 × 8 W bulbs, output from ~300–460 nm, peak at 365 nm), and the reaction mixture was left in the molds overnight. The hydrogels were left in DI water for 24 h to remove unreacted monomers and low molecular weight contaminants with periodic changes of water (typically 3 to 4 times per day), after which they were transferred to fresh water for another 24 h.

#### 2.1.3. Preparation of Conductive Hydrogels

A solution of modified CS in DI water (0.45 wt%) was stirred until homogenous. The photoinitiator 2-hydroxy-4′-(2-hydroxyethoxy)-2-methylpropiophenone (Irgacure-2959) was added at 0.1 wt% to the solution of CS derivative; and PEG-diacrylate (PEGDA, Mn 2000) was added at 5 wt%. Pyrrole (200 μL, 4.3 mmol per mL of PEGDA-modified CS) was purified by passage through an alumina column added to 1 mL of PEGDA-modified CS with lignin (lignin alkali, low sulfonate content, 100 mg/mL), and this mixture was stirred in the dark for ca. 2 h until homogenous. Silicone isolators (molds) produced by Grace Bio-Labs (Sigma Aldrich) (9 mm in diameter × 0.8 mm in depth) were placed onto clean glass slides. 75 μL of gel precursor solution was added to each well and irradiated for 1 h under a Mega LV202E UV exposure unit (with 2 × 8W bulbs, output from ~300–460 nm, peak at 365 nm), and the reaction mixture was left in the molds overnight. Ferric chloride (FeCl_3_, 1 wt%) was dissolved in water and the gels were left to incubate for 24 h, after which the supernatant was decanted and the samples were washed thoroughly with DI water until the water became clear and colorless (typically 72 h to remove unreacted monomers and low molecular weight contaminants with periodic changes of water, typically 3 to 4 times per day), after which they were transferred to fresh water for another 24 h.

### 2.2. Characterization

#### 2.2.1. UV-Vis Spectroscopy

UV-vis spectra were recorded using a Thermo Scientific NanoDrop 2000. CS was dissolved in an aqueous solution of acetic acid (1% *v*/*v*) whereas the other samples were in DI water.

#### 2.2.2. Attenuated Total Reflectance Fourier Transform Infrared (ATR-FTIR) Spectroscopy

An Agilent Technologies Infrared Spectrometer was used to record spectra in ATR mode in the range of 4000–500 cm^−1^. The data was exported to the software supplied by the manufacturer (ResPro) and the baseline was corrected.

#### 2.2.3. Solution State ^1^H NMR Spectroscopy

^1^H NMR spectra were recorded with a Bruker AVANCE III 400 (NanoBay) equipped with a 5 mm ^1^H-X broadband observe (BBO, ^109^Ag-^19^F) RT probe, and (3-trimethylsilyl)propionic-2,2,3,3-d_4_ sodium salt was added as a reference in all samples. 5 mm NMR tubes (Norell standard series 5) were used for all samples. 1 mg of CS medium MW was dissolved in 1 mL of 2% DCl *v*/*v* (35 wt% in D_2_O) in D_2_O solution and heated to ~70 °C for an hour or until complete dissolution. For modified CS, the same procedure was followed however heating was not necessary. Acetic acid was dissolved in D_2_O at a concentration of (5 mg/mL). Pyrrole-2-carboxylic acid was dissolved in DMSO-d_6_ at a concentration of (5 mg/mL). The spectra of methacrylic acid was acquired in both D_2_O and DMSO-d_6_.

#### 2.2.4. Solid State ^13^C NMR Spectroscopy

A Bruker AVANCE III HD 700 WB was used to record ^13^C NMR spectra via cross-polarization/magic angle spinning.

#### 2.2.5. Scanning Electron Microscopy (SEM)

Prior to imaging the samples were lyophilized (FreeZone benchtop freeze dryer, Labconco™, Fisher Scientific, Loughborough, UK), then sputter coated with a 10 nm layer of gold using a Quorum 150R ES. A JEOL JSM-7800F field emission SEM (JEOL, Welwyn Garden City, UK) operating at 10–15 kV was used to obtain SEM images.

#### 2.2.6. Conductivity Determination

The conductance of dried gels were measured in accordance with protocol IPC-TM-650, number 2.5.17.2 described by the Institute for Interconnecting and Packaging Electronic Circuits. Dried gels supported on glass slides were examined by chronoamperometry using a Keithley 2612B source meter (Tektronix, Beaverton, OR, USA). Chronoamperometric measurements were made with a two-point probe system (copper alligator clips), by connecting counter and reference electrodes together. Briefly, two thin strips of adhesive-backed copper tape (Ted Pella, Inc., Redding, CA, USA) were attached to the dried gels, parallel to one another, separated by a distance of 0.5 cm. The working and counter electrodes were clipped on the strips of copper tape, and the current measured for 30 s during a potential step experiment at 10 V. The electrodes were moved to different positions after each measurement, and the current passed was recorded at three different positions. The resistance (*R*, Ω) of the gels was determined by measurements of voltage (*V*) and current (*I*) in accordance with Equation (1):

*R = V/I* (1)

The resistivity (Ω cm^−1^) of the dried gels was determined in accordance with Equation (2):

(2) ρ=Rwt/L

In which: *w* corresponds to the width of the gel in cm (ca. 0.5 cm, determined on a sample-by-sample basis using digital calipers, Scienceware^®^ Digi-Max™ slide calipers); *t* corresponds to the thickness of the dried gel in cm (as determined via profilometry); and *L* corresponds to the length of the gel in cm (ca. 0.5 cm, determined on a sample by sample basis using digital calipers). The conductivity (S cm^−1^) of the gels was determined in accordance with Equation (3):

(3) σ=1/ρ

#### 2.2.7. X-ray Diffraction (XRD)

XRD patterns were recorded using a Rigaku SmartLab powder diffractometer with a 2θ scattering range of 5 to 40° and a resolution of 0.1°.

#### 2.2.8. Thermogravimetric Analysis (TGA)

Thermal stability of vacuum dried hydrogels (ca. 4–5 mg) were observed with a NETZSCH STA 449 F3 Jupiter thermal analyser. Temperatures observed were from 25 to 550 °C with a heating rate of 10 °C/min. The reference pan used was alumina.

#### 2.2.9. Swelling Studies

The swelling ratio of the hydrogels was determined by recording the initial mass its dry state and the mass after swelling in PBS for 24 h (after removal of excess water by wicking with filter paper) at room temperature, and calculated using Equation (4), where *m_t_* is the mass at the time *t* and *m*_0_ represents the initial mass.

(4) $$Swelling\;Ratio\;\left(\%\right)=\frac{\left(m_{t}-m_{0}\right)}{m_{0}}\times 100$$

#### 2.2.10. Rheological Characterization

The rheological properties of the gels were assessed using an Anton Paar Physica MCR 302 rheometer fitted with a parallel plate with a diameter of 12.484 mm. Strain sweep experiments were employed with a constant frequency of 10 rad/s. Frequency sweeps were performed at 0.5% strain for non-conductive hydrogels and at 0.1% for conductive hydrogels.

### 2.3. In Silico and In Vitro Validation

#### 2.3.1. In Silico Toxicity Screening Studies

In silico toxicity screening was carried out using Derek Nexus and Sarah Nexus (Derek Nexus: v. 6.0.1, Nexus: 2.2.2; Sarah Nexus: v. 3.0.0, Sarah Model: 2.0) supplied by Lhasa Limited, Leeds, UK. The SMILES code for Irgacure-2959 is C(COC1=CC=C(C=C1)C(C(C)(C)O)=O)O; for a simplified PEG-diacrylate it is C=CC(OCCOC(C=C)=O)=O; and for the modified CS derivative it is O7[C@@H](O[C@@H]5C(O[C@@H](O[C@@H]1C(O[C@H](C(C1O)N)O[C@@H]4C(O[C@@H](O[C@@H]2C(O[C@H](C(C2O)N)O[C@@H]3C(O[C@@H](O)C(C3O)N)CO)CO)C(C4O)NC(C(C)=C)=O)CO)CO)C(C5O)NC(C6=CC=CN6)=O)CO)C(C([C@@H](C7CO)O)O)N.

#### 2.3.2. In Vitro Cell Culture Studies

The NIH3T3 fibroblast cell line was cultured in Dulbecco’s Modified Eagle’s Medium (DMEM) with 10% fetal calf serum, 1% penicillin–streptomycin and 0.25 μg/mL Fungizone, under an atmosphere of 5% CO_2_ at 37 °C. Cells were harvested using a trypsin–EDTA solution and the number of viable cells was counted with a Neubauer camera after staining with trypan blue. Fibroblast cells (1 × 10^4^ cells per well) were added on top of each material or to the wells of 24-well plate (in case of control cells), with 0.5 mL of cell culture medium and kept in a humidified 5% CO_2_ atmosphere at 37 °C. The cell metabolic activity was measured by the colorimetric 3-(4,5-dimethyl-thiazol-2-yl)-2,5-diphenyl-tetrazolium bromide (MTT) assay after 1–7 days. Briefly, cell culture medium was removed and a solution of MTT in PBS 1X (5 mg/mL) was added to the cells and incubated at 37 °C for 4 h. Then, the MTT solution was discarded, samples were washed with PBS 1X and absolute ethanol was added. Finally, absorbance of the purple solution was measured at 570 nm and results were expressed as mean ± SD from triplicate experiments. For cell imaging, cells grown for 24 h were first fixed using 2.5% glutaraldehyde. After 20 min, samples were washed three times with PBS 1X and 0.1% Triton X-100 in PBS 1X containing 1% BSA was added to permeabilize the cells for 30 min. Samples were then washed with PBS 1X and 300 μL of Alexa 488 phalloidin were added for staining. Cells were incubated at 37 °C and protected from light for 30 min. After this, samples were washed with PBS 1X and the phalloidin-stained cells were finally examined by fluorescence microscopy using a 20X objective on an inverted microscope (Confocal—Zeiss—AxioObserver Z1 LSM710) [76,77,78].

#### 2.3.3. In Vitro Drug Loading

Conductive hydrogels were electrochemically loaded with pemetrexed using an aqueous solution of 4 mL of 1 mM pemetrexed disodium 2.5 hydrate as the electrolyte bath. The hydrogels were secured on glassy carbon electrodes by the electrode lid with a hole cut out at the top (Figure S1). A 3 electrode cell was composed of a Ag/AgCl reference electrode, platinum mesh counter electrode and a glassy carbon electrode with the hydrogel. For 30 min a potential of 0.6 V was applied via chronoamperometry. 10 μL of the solution after drug loading was diluted down 4 times with distilled water and the concentration of PEM assessed via UV-Vis measurements at 225 nm (λ_max_ of PEM) from which the loading efficiency is assessed and the difference in the concentration of PEM before/after used to assign “100%” loading.

#### 2.3.4. In Vitro Drug Delivery Studies

UV spectroscopy confirmed that there was no release of the gel constituents from the unloaded gels (e.g., lignin, modified CS, PPy) over the course of the experiments.

For stimulated drug release, the drug was released at a potential of −0.6 V in 4 mL of PBS for 30 s every 10 min 3 times. At each time interval, 10 μL of the solution was frozen for storage prior to UV-Vis spectroscopy.

Passive release of the drug was carried out by placing the electrodes with hydrogels in 4 mL PBS and 10 μL samples were taken every 11 min in line with the sampling frequency for the samples that were electrically stimulated.

At each time interval, 10 μL of the solution was frozen for storage prior to UV-Vis spectroscopy. The concentration of PEM assessed via UV-Vis measurements at 225 nm (λ_max_ of PEM).

## 3. Results and Discussion

### 3.1. CS Modification and Characterization

**Scheme 1:**
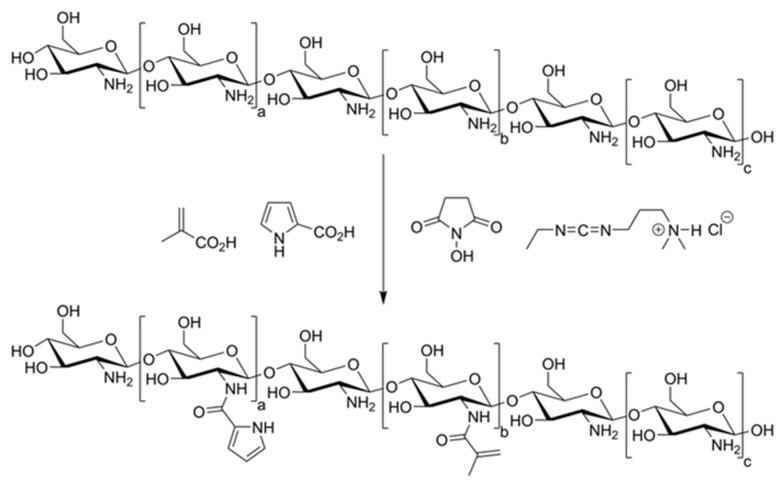
Chemical modification of CS (conjugation of methacrylate and pyrrole moieties via EDC/NHS chemistry).

Methacrylic acid and pyrrole-2-carboxylic acid were conjugated to the amines displayed on the backbone of CS (a white/off-white solid in the dry state) using water soluble carbodiimide (EDC/NHS) chemistry, after dialysis and lyophilization this yielded a pink/purple solid (Scheme 1).

**Figure 1:**
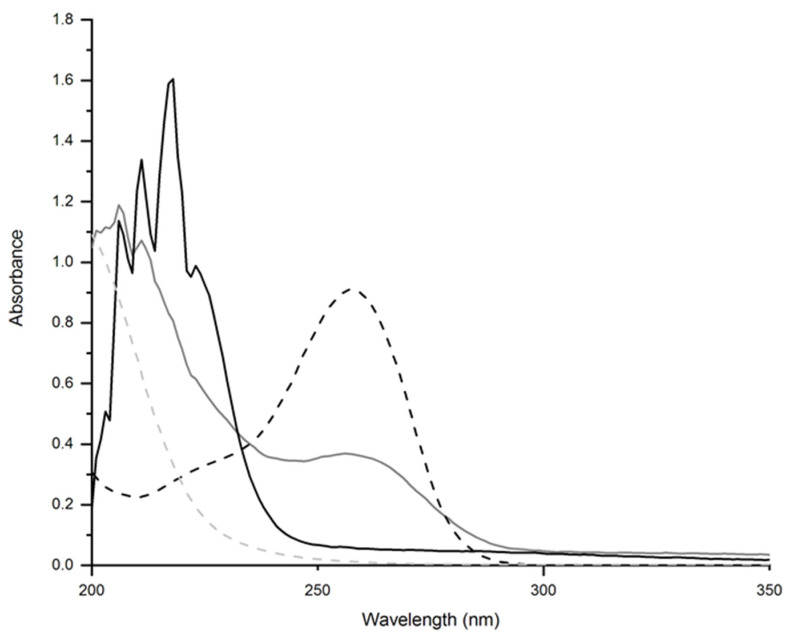
UV-Vis spectra of CS (solid black line), modified CS (grey line), pyrrole carboxylic acid (black dashed) and methacrylic acid (grey dashed).

The UV-vis spectra of the starting materials and product are displayed in Figure 1; clear differences between the spectra can be identified, while CS has a maxima of 218 nm (owing to N-acetylglucosamine (GluNAc) and glucosamine (GlcN)), the modified CS has a broad peak at around 195 nm with two peaks at ~200 and 255 nm that are not present in the CS starting material which are attributed to the methacrylate and pyrrole moieties conjugated to the CS.

**Figure 2:**
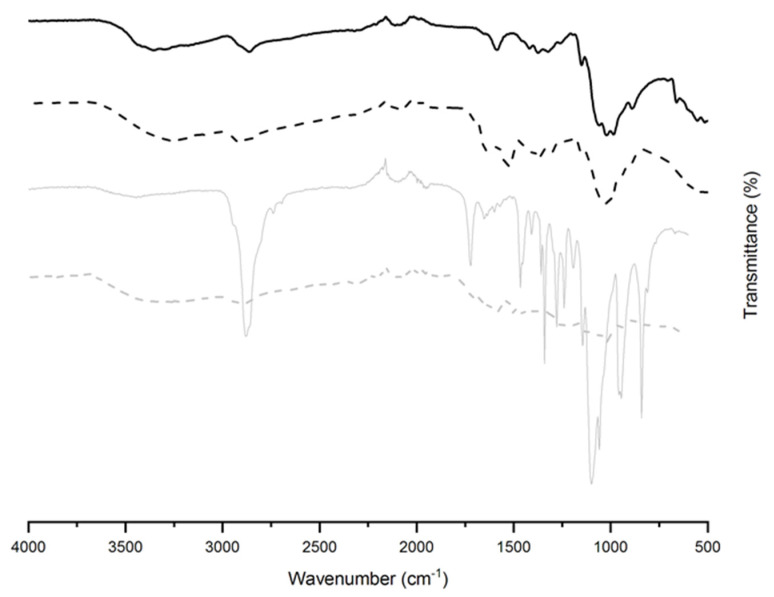
FTIR spectra. Unmodified CS (black solid line); modified CS (black dashed line); non-conductive hydrogel (grey solid line); conductive hydrogel (grey dashed line).

The FTIR spectra for the CS starting material and the modified CS are shown in Figure 2. CS shows a broad peak at 3354 cm^−1^ which corresponds to -OH stretching overlapping with -NH_2_ symmetric and asymmetric stretching bands; peaks occurring at 1649, 1527 cm^−1^ correspond to the amide I (C=O stretch) and amide II (N-H bend, C-N stretch) vibrations, respectively; the absorbance at 1566 cm^−1^ corresponds to the N-H bend of an amine group (i.e., deacetylated from chitin); multiple low intensity peaks at 1419, 1373, 1321 cm^−1^ are ascribed to C-H bending; the peak at 1149 cm^−1^ is characteristic of C-O-C ether bonds present in the backbone of CS; the sharp absorbances at 1020, 1057 cm^−1^ are C-O stretches in the alcohol groups. The spectrum of the modified CS shows increases in the intensity of the amide I, amide II peaks, and those ascribed to C-H bending and stretching confirming modification with methacrylate/pyrrole units. The spectrum for the conductive samples were very broad due to experimental limitations of the technique, including: optical contact between the sample and ATR apparatus, anomalous dispersion [79,80,81], and the fact they are broad band absorbers which resulted in broad spectra as we have observed in previous studies [82].

**Figure 3:**
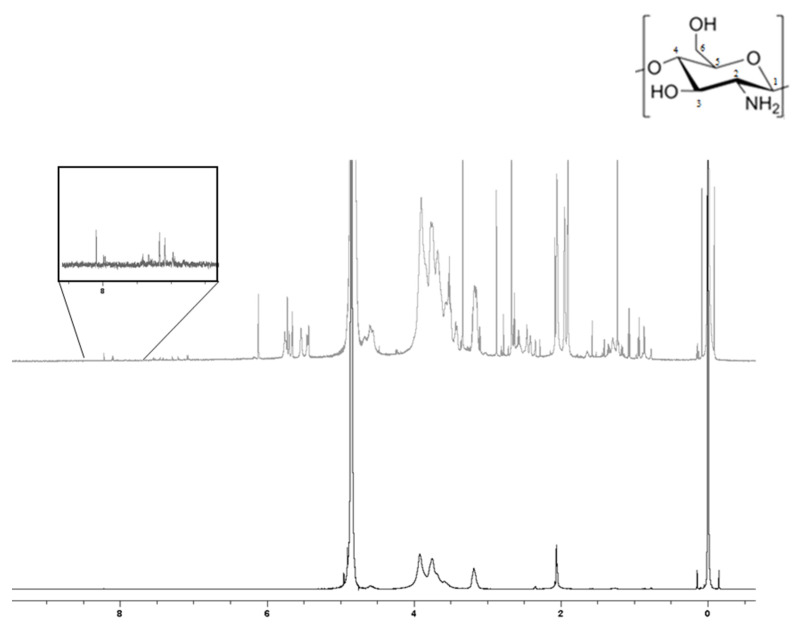
^1^H solution state NMR spectra. (**Bottom**) Unmodified CS. (**Top**) Modified CS.

**Figure 4:**
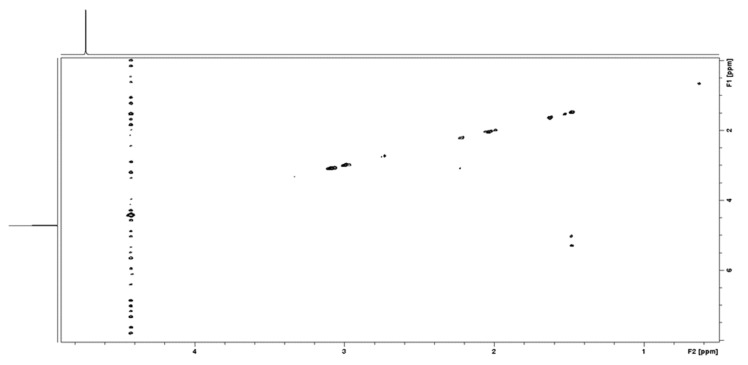
^1^H-^1^H COSY solution state NMR spectra of modified CS.

Solution state ^1^H NMR spectra for the CS starting material and the modified CS derivative in DCl/D_2_O are shown in Figure 3. The small peak at around 2.03 ppm is ascribed to the methyl group (-CH_3_) attached to the N-alkylated GlcN residue. At 3.10 ppm a singlet represents H2, multiplet signals from 3.5 to 4 ppm are assigned to H3, H4, H5 and H6 of GlN. From the literature, typically the spectra would show a small peak around 4.28 ppm due to H1 of both GlN and the acetylated form [83,84]. Comparison of the peak integrals from H2-H6 (~3 to 4 ppm) which represent 6 protons to the integral of the peak at 2.03 ppm which represents 3 protons enables estimation of the degree of acetylation of the CS (the partially deacetylated derivative of chitin) which was ca. 12% in this study, i.e., the unmodified CS used is around 88% deacetylated (similar to the supplier’s estimate of 75–85% deacetylation). The spectra for modified CS shows new chemical shifts corresponding to the new functional groups installed. The peak at 1.9 ppm is characteristic of the methyl group attached to the methacrylate (confirmed by the NMR spectra of methacrylic acid showing a peak at 1.9 ppm). Signals from ~5 to 6.5 ppm are assigned to the two protons connected to the double bond. A multiplet of peaks with low intensity can be seen between 7.9 and 8.1 ppm which are ascribed to pyrrole attached to the backbone of CS (albeit very difficult to integrate in comparison to methacrylate moieties). Two-dimensional ^1^H—^1^H COSY NMR links methacrylate olefins (5.5–6 ppm) to the methyl at 1.9 ppm (Figure 4).

**Figure 5:**
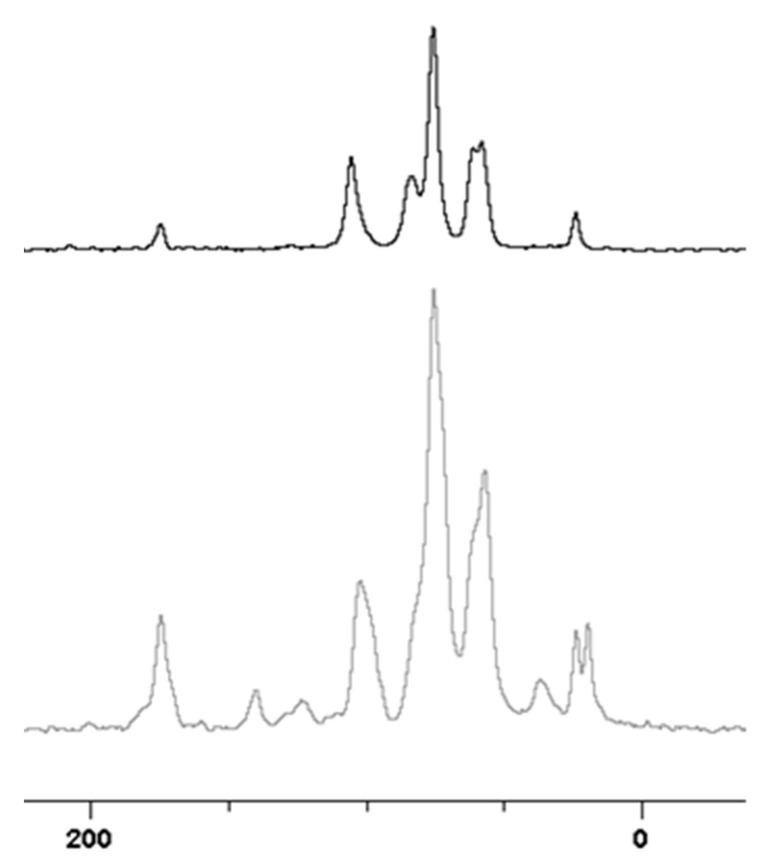
^13^C solid state NMR spectra. (**Top**) Unmodified CS; (**Bottom**) Modified CS. N.B. X-axis in ppm.

Solid State ^13^C NMR CP-MAS spectra were recorded. The spectrum of unmodified CS starting material is shown in Figure 5, and the chemical shifts in accordance with the literature [85,86,87,88,89,90,91,92,93]. The modified CS exhibits similar chemical shifts to CS, signals at 105, 83, 75, 61 and 58 ppm correspond to C1, C4, C3, C6, C2 (Figure 5); the peaks in both spectra corresponding to C=O and CH_3_ (due to partial deacetylation and amide) appear at 174 and 24 ppm, respectively; the conjugation of pyrrole was confirmed by peaks for the α-carbon and β-carbon at ca. 125 and 109–105 ppm [94,95,96,97], and previous research by Forsyth et al. explains the low frequency shoulder on the peak at 123 ppm exists due to β-carbons being partially oxidized as opposed to pure pyrrole [94,95,96,97]; the conjugation of methacrylate was confirmed by peaks 19 ppm (CH_3_), 37 ppm (C-C tertiary carbons pendant to the polymer chain) and 140 ppm (C=C) in line with the literature [98]. The peak for the carbonyls (C=O) pendant on the polymer chain for the unmodified CS (ca. 175 ppm) was significantly broader after conjugation of the methacrylate and pyrrole derivatives (ca. 170–180 ppm) owing to the multitude of different amide environments generated by the conjugation reaction in line with the literature [99,100].

### 3.2. Hydrogel Preparation and Characterization

Non-conductive hydrogels composed of PEG and CS were prepared by photopolymerization of PEGDA and the modified CS derivative in an aqueous solution of the photoinitiator Irgacure-2959, followed by washing. Conductive hydrogels composed of PEG, CS, PPy and lignin were prepared by photopolymerization of PEGDA, the modified CS derivative, pyrrole and lignin in an aqueous solution of the photoinitiator Irgacure-2959, followed by exposure of the gels to FeCl_3_ to polymerize the pyrrole (followed by extensive washing to remove low molecular weight species), yielding PPy doped with anionic lignin and chloride distributed throughout the gels an interpenetrating polymer network; the resultant self-supporting gels were black colored due to the presence of the PPy and lignin. SEM images (Figure S2) of the surfaces of non-conductive hydrogels are smooth on the μm scale by comparison with the conductive hydrogels due to the particles of lignin-doped PPy embedded within the conductive gels (the PPy particles were also observed to be distributed throughout the gel matrix as a sample spanning interpenetrating network of PPy in cross-sectional images). The conductance of dried gels was observed to be of the order of 2.65 × 10^−9^ S/cm for the dried CS-PEG gels, and 8.88 × 10^−7^ S/cm for the PPy containing gels, similar, albeit somewhat lower than analogous PPy-containing materials [74,101,102], which is likely due to the high content of PEG and lignin in these materials.

The FTIR spectra for the non-conductive and conductive gels are shown in Figure 2. For the non-conductive gels, in addition to the peaks associated with the modified CS, the peaks associated with PEG (the major component of the non-conductive gels) are notable, with a strong band between 2960–2770 cm^−1^ from C-H stretching in alkanes/ethers, a peak at ca. 1740 cm^−1^ corresponding to the C=O stretch of the ester bonds that attach the methacrylates to the termini of the PEG chains, and the very strong peak at 1149 cm^−1^ is characteristic of C-O-C ether bonds present in the backbone of PEG. The spectrum of conductive gels is different from the non-conductive gels, and all peaks are notably broader which is representative of the complex nature of the interpenetrating network of different polymeric species (PEG, CS, PPy and lignin). XRD patterns of the non-conductive gels and conductive gels suggested they were amorphous, confirmed by the peak at 2θ ≈ 23° for the non-conductive gels and at 2θ ≈ 25° for the conductive gels (Figure S3). TGA of dried gels (Figure S4) confirmed the presence of traces of water in the gels (mass loss between 45–180 °C). The non-conductive hydrogels began to degrade at ca. 200°C, and the majority of mass occurred between 200 °C and 380 °C in line with the literature [103]; by comparison, the conductive hydrogels degraded between 260 °C and 500 °C in line with the literature [104], implying the interpenetrating network enhances thermal stability. The TGA profiles suggest the compositions of the conductive gels to be ca. 30% PEG/CS and 70% PPy/lignin.

In principle this may enable, sterilization by steam in an autoclave at 121 °C for 20 min, although there may be consequences for the mechanical/electrical properties of the hydrogels [105] and optimization of sterilization will be dependent upon the specific application of the hydrogels. The swell ratios of the non-conductive gels were ca. 1029 ± 327% whereas for the conductive gels it was ca. 123 ± 25%, suggesting that the interpenetrating network of anionic lignins and cationic CS/PPy in the conductive gels serve to further crosslink the gels.

**Figure 6:**
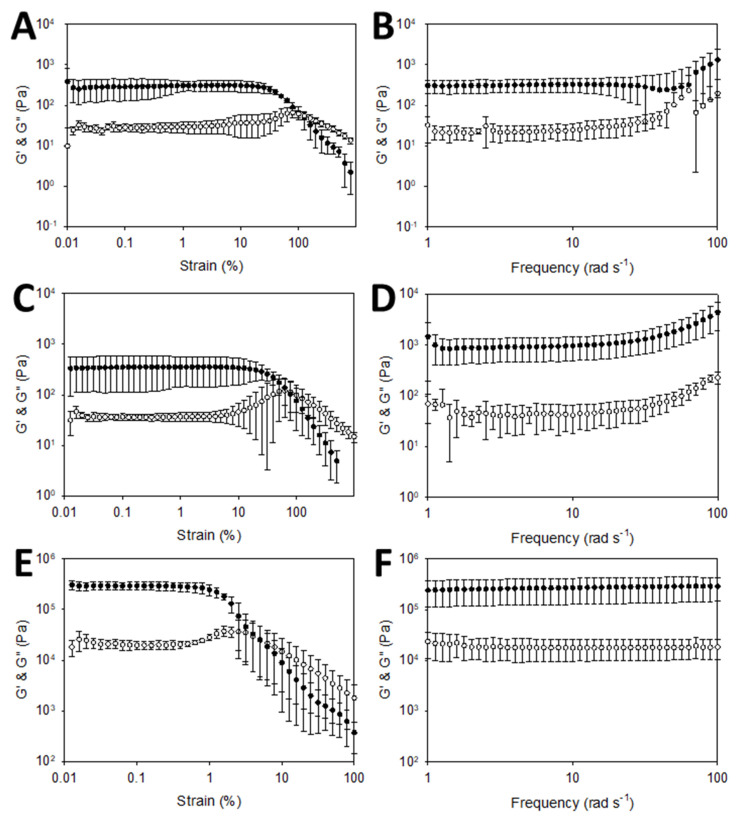
Rheology data. (**A**) strain sweep: non-conductive hydrogels (wet). (**B**) Frequency sweep: non-conductive hydrogels (wet). (**C**) strain sweep: conductive hydrogels (dry). (**D**) Frequency sweep: non-conductive hydrogels (dry). (**E**) strain sweep: conductive hydrogels (wet). (**F**) Frequency sweep: conductive hydrogels (wet).

The rheological properties of the hydrogels were characterized by both dynamic strain sweep testing (performed at a constant frequency of 1Hz (10 rad/s)) and frequency sweep testing (performed at 0.5% strain for non-conductive hydrogels and at 0.1% for conductive hydrogels) to assess the storage modulus (G′—elastic) and loss modulus (G″—viscous) of the hydrogels (Table 2 and Figure 6). When G′ is higher than G″ it suggests that the gel is highly structured and the material behaves in a solid-like fashion; the point where both moduli intersect signifies the breaking point of the hydrogels and is known as the critical strain level or gel point; as strain increases past the critical strain level, the network bonds start to break and the moduli both start to decline (when G′ drops below G″ the gel is more liquid-like) [106]. The properties for the non-conductive and conductive gels are summarized in Table 2.

The non-conductive gels are soft (G′ 0.35 kPa, G″ 0.02 kPa), with properties analogous to brain tissue; the conductive gels in the dry state are approximately twice as strong as the non-conductive hydrogels, and in the wet state the conductive gels are significantly stronger (G′ 30 kPa, G″ 19 kPa) analogous to breast tissue [107]. The differences in mechanical properties are likely due to intermolecular interactions between the interpenetrating network of anionic lignins and cationic CS/PPy in the conductive gels.

### 3.3. In Silico and In Vitro Validation

We have previously examined the safety data sheets for (FeCl_3_, pyrrole and polpyrrole) which report FeCl_3_ to be a corrosive irritant that is toxic to aquatic life, and pyrrole is corrosive and could be toxic if swallowed (necessitating thorough washing to remove the FeCl_3_ and pyrrole), however, PPy and PSS are nontoxic; and employed in silico toxicity screening (Derek Nexus and Sarah Nexus) which demonstrated they were non-sensitizers of skin and non-mutagenic [101,108,109]. Here, we extend this approach to assessing the safety of the other components in the gels. The safety data sheets for unmodified CS and lignin suggest they are non-hazardous, whereas the PEG-diacrylate is a skin irritant/sensitizer and can cause serious eye damage, and the Irgacure-2959 may be toxic (LD50 Oral—Rat > 2000 mg/kg; LD50 Dermal—Rat [male and female] > 5000 mg/kg) [100,101], necessitating thorough washing to remove them after gel formation. Derek Nexus predicts that: Irgacure-2959 is non-mutagenic in vitro in bacterium (supported by Sarah Nexus) and a non-skin sensitizer in mammals; the modified CS is non-mutagenic in vitro in bacterium (supported by Sarah Nexus) and a plausible skin sensitizer and irritant in mammals (however, these are similar to unmodified CS, i.e., not due to the new functional groups attached), however, the new functional groups do render the modified CS derivative plausibly neurotoxic due to the acrylamide present after methacrylation [108,109,110,111,112,113,114,115]; the PEG-diacrylate is non-mutagenic in vitro in bacterium (supported by Sarah Nexus) and a plausible skin sensitizer and irritant in mammals, a plausible irritant of eyes/respiratory tracts in mammals, plausibly causes chromosome damage in vitro in mammals and unclear if it is carcinogenic in mammals. Clearly, as noted above the safety data sheets and in silico toxicity screening emphasize the necessity for thorough washing of the gels after production to remove any low molecular weight contaminants.

**Figure 7:**
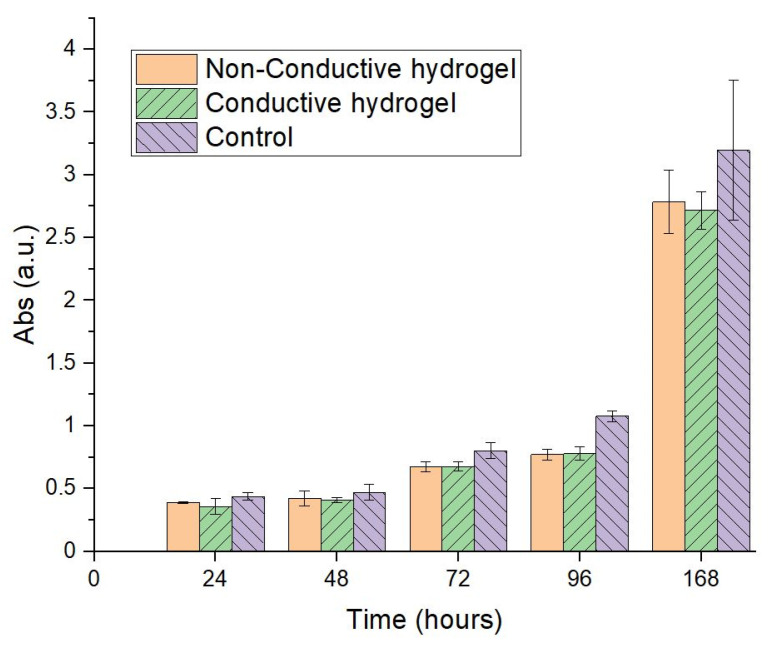
Cell adhesion/proliferation data.

In vitro studies to assess the adhesion and proliferation of NIH3T3 fibroblasts on the hydrogels were conducted, using the standard 3-(4,5-dimethylthiazol-2-yl)-2,5-diphenyltetrazolium bromide (MTT) assay was used to study cell adhesion and proliferation over the period of one week compared to the cells on tissue culture plastic, assessed at 1, 2, 3, 4 and 7 days. NIH3T3 fibroblasts adhered and proliferated on the non-conductive and conductive gels (no discernable differences between the data for the types of gel), albeit slightly less effectively on the gels than on the tissue culture plastic control (Figure 7).

**Figure 8:**
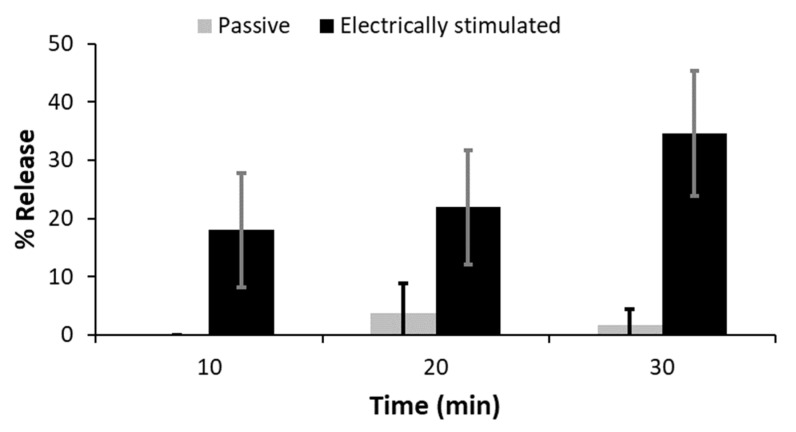
Drug release profiles of hydrogels loaded with PEM. Without electrical stimulation (grey bars) and electrochemically triggered (black bars).

We studied the release of PEM from PEM-doped hydrogels into phosphate-buffered saline (PBS) in the absence/presence of electrical stimuli via UV spectroscopy (Figure 8) over the period of 30 min, a duration chosen as it is used in treatment of cancer via electrochemotherapy [59,60,66]. Passive release of PEM was observed from all hydrogels, however, this amounted to less than 5% over the course of the experiment, and the application of an electrical stimulus was observed to trigger the delivery of PEM from the gels, with an increase of ca. 10–15% relative to the passive release control experiment for each application of electrical stimulation. This triggered release of PEM over the 30 min duration would be potentially useful for surgical procedures which may benefit from localized delivery of anticancer drugs, for example integration of these hydrogels as coatings on electrodes used for electrochemotherapy [74,75].

The conductive hydrogels described herein are straightforward to prepare, with 1 synthetic step for the modified CS derivative, and a simple gel preparation protocol involving a photopolymerization step, followed by an oxidative polymerization step, followed by washing to remove non-crosslinked components. We foresee that the gel formulations and the protocols described could be adapted for 3D printing of bespoke 3D structures [116,117,118] with mechanical properties suitable for integration within a variety of soft human tissues [107]. It would be possible to incorporate and release a variety of different bioactive molecules with wide ranging molecular weights (including low molecular weight pharmaceuticals and high molecular weight biologics) depending on the specific tissue in which the biomaterials would be integrated [119,120]. The opportunity to precisely control the delivery of a payload is potentially very impactful where the chronobiology of the condition being treated is important [121,122].

## 4. Conclusions

The electroactive hydrogels described herein were prepared by a simple methodology, were robust enough to be handleable, and it is foreseeable they could be integrated into medical devices for the delivery of clinically relevant drugs. Such smart drug delivery systems represent a significant opportunity for patient-specific treatments in the future.

## Figures and Tables

**Table 1:** Formulations used for the preparation of non-conductive and conductive gels.

Component	Non-Conductive Gels	Conductive Gels
Water	1 mL	1 mL
PEGDA	50 mg	50 mg
Modified CS derivative	4.5 mg	4.5 mg
Lignin	N/A	100 mg
Pyrrole	N/A	200 μL

**Table 2:** Rheological properties of the non-conductive and conductive gels.

Variable	Non-Conductive Gels (Wet)	Conductive Gels (Dry)	Conductive Gels (Wet)
G′	350 Pa	650 Pa	30,000 Pa
G″	22 Pa	54 Pa	19,000 Pa
Yield point	15%	7%	0.8%
Flow point	95%	140%	4%

## Data Availability

Data available on reasonable request to the authors.

## Supplementary Materials

The following supporting information can be downloaded at: https://www.mdpi.com/article/10.3390/polym14224953/s1, Figure S1: Side view (left) and top view (right) of set up for securing hydrogels on glassy carbon electrodes for drug delivery studies; Figure S2: SEM images (×1000 magnification). Top) non-conductive gels. Bottom) conductive gels. Scale bars represent 10 μm; Figure S3: X-ray diffractograms. Non-conductive gels (grey line); conductive gels (black line); Figure S4: TGA thermograms. Non-conductive hydrogels (grey); conductive hydrogels (black).
