# Supplementary material for: Electrochemically Enhanced Delivery of Pemetrexed from Electroactive Hydrogels
Source: Polymers (Basel). 2022 Nov 16;14(22):4953. doi: 10.3390/polym14224953 (PMC9692448; doi:10.3390/polym14224953)
Supplement: Supplementary file 1 [file polymers-14-04953-s001.zip › polymers-1952050-supplementary.pdf]

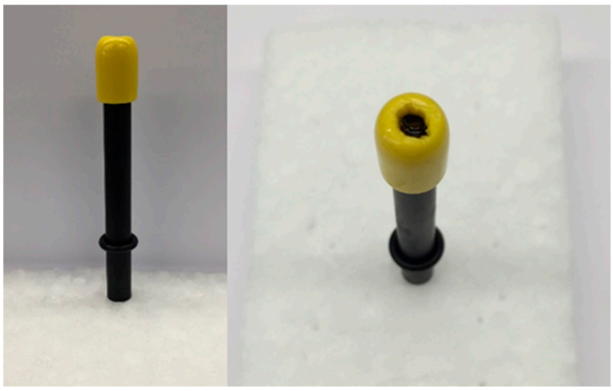

**Figure S1.** Side view (left) and top view (right) of set up for securing hydrogels on glassy carbon electrodes for drug delivery studies.

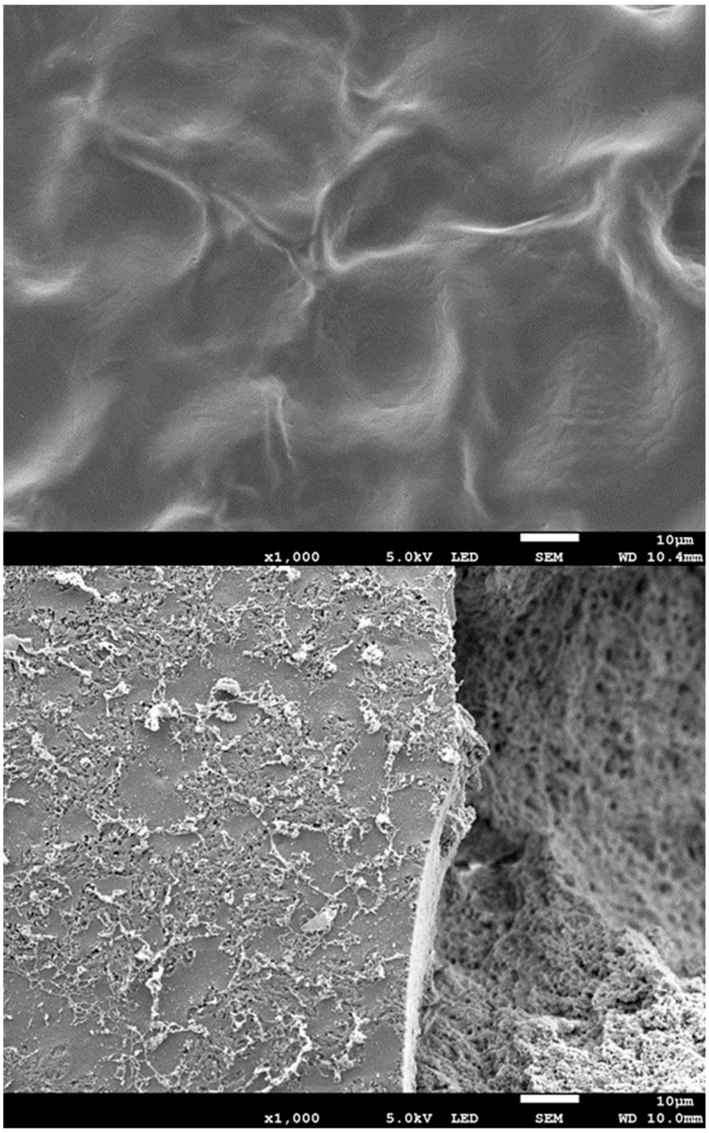


**Figure S2.** SEM images (x1,000 magnification). Top) non-conductive gels. Bottom) conductive gels. Scale bars represent 10 µm.

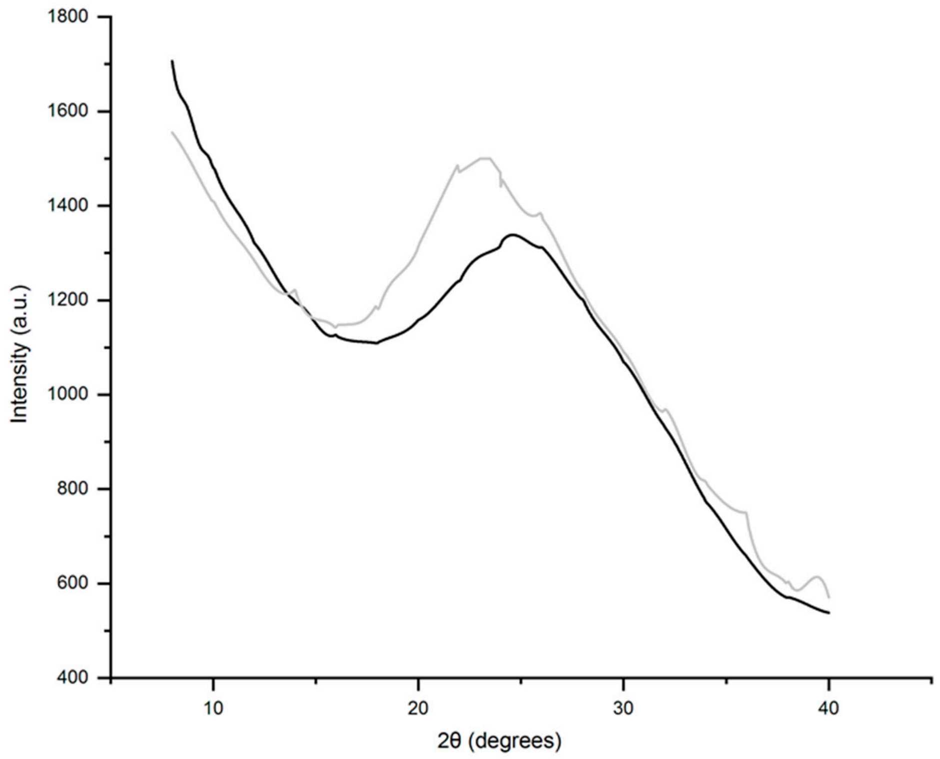


**Figure S3.** X-ray diffractograms. Non-conductive gels (grey line); conductive gels (black line).

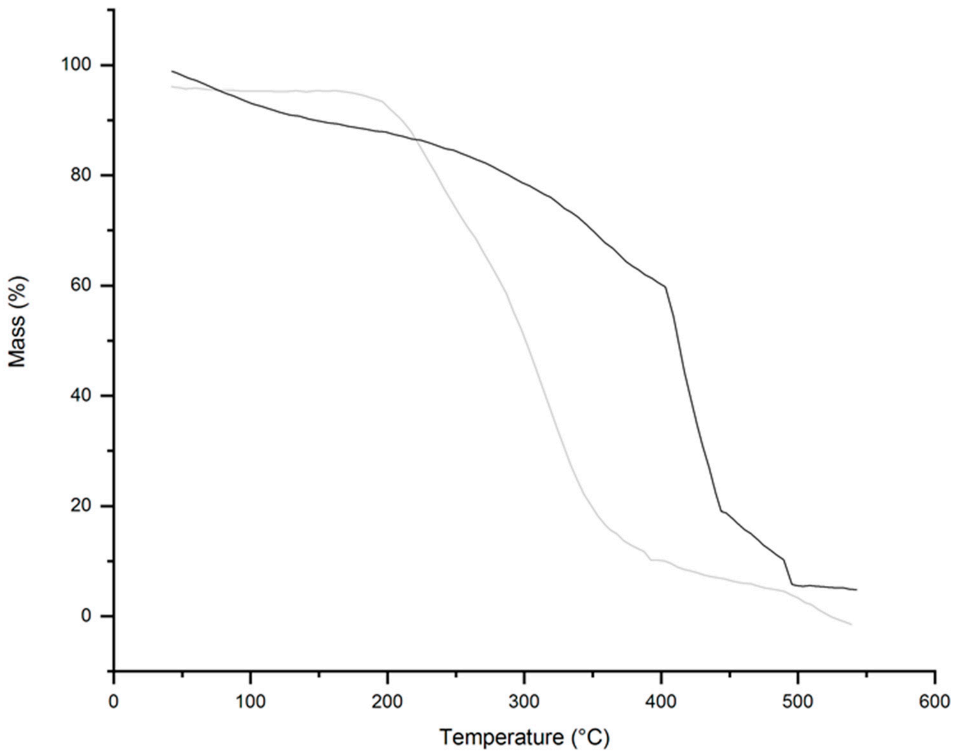


**Figure S4.** TGA thermograms. Non-conductive hydrogels (grey); conductive hydrogels (black).
